# Phenotypic and genotypic monitoring of *Schistosoma mansoni* in Tanzanian schoolchildren five years into a preventative chemotherapy national control programme

**DOI:** 10.1186/s13071-017-2533-6

**Published:** 2017-12-02

**Authors:** Charlotte M. Gower, Florian Gehre, Sara R. Marques, Poppy H. L. Lamberton, Nicholas J. Lwambo, Joanne P. Webster

**Affiliations:** 1 Centre for Endemic, Emerging and Exotic Diseases, The Royal Veterinary College, University of London, London, AL9 7TA UK; 2 Department of Infectious Disease Epidemiology, Imperial College, Faculty of Medicine, W2 1PG, London, UK; 3 Mycobacteriology Unit, Institute of Tropical Medicine, Antwerp, Belgium; 4 Department of Life Sciences, Imperial College, Faculty of Medicine, London, UK; 5 Institute of Biodiversity, Animal Health & Comparative Medicine & Wellcome Centre for Molecular Parasitology, University of Glasgow, G12 8QQ, Glasgow, UK; 6 Mwanza Research Centre, National Institute for Medical Research, Mwanza, Tanzania

**Keywords:** Density-dependence, Neglected tropical diseases, Schistosomiasis, Population genetics, Drug resistance, Praziquantel

## Abstract

**Background:**

*Schistosoma mansoni* is a parasite of profound medical importance. Current control focusses on mass praziquantel (PZQ) treatment of populations in endemic areas, termed Preventative Chemotherapy (PC). Large-scale PC programmes exert prolonged selection pressures on parasites with the potential for, direct and/or indirect, emergence of drug resistance. Molecular methods can help monitor genetic changes of schistosome populations over time and in response to drug treatment, as well as estimate adult worm burdens through parentage analysis. Furthermore, methods such as in vitro drug sensitivity assays help phenotype in vivo parasite genotypic drug efficacy.

**Methods:**

We conducted combined in vitro PZQ efficacy testing with population genetic analyses of *S. mansoni* collected from children from two schools in 2010, five years after the introduction of a National Control Programme. Children at one school had received four annual PZQ treatments and the other school had received two mass treatments in total. We compared genetic differentiation, indices of genetic diversity, and estimated adult worm burden from parasites collected in 2010 with samples collected in 2005 (before the control programme began) and in 2006 (six months after the first PZQ treatment). Using 2010 larval samples, we also compared the genetic similarity of those with high and low in vitro sensitivity to PZQ.

**Results:**

We demonstrated that there were individual parasites with reduced PZQ susceptibility in the 2010 collections, as evidenced by our in vitro larval behavioural phenotypic assay. There was no evidence, however, that miracidia showing phenotypically reduced susceptibility clustered together genetically. Molecular analysis also demonstrated a significant reduction of adult worm load over time, despite little evidence of reduction in parasite infection intensity, as measured by egg output. Genetic diversity of infections did not reduce over time, despite changes in the genetic composition of the parasite populations.

**Conclusions:**

Genotypic and phenotypic monitoring did not indicate a selective sweep, as may be expected if PZQ treatment was selecting a small number of related “resistant” parasites, but there was evidence of genetic changes at the population level over time. Genetic data were used to estimate adult worm burdens, which unlike parasite infection intensity, showed reductions over time, suggesting the relaxation of negative density-dependent constraints on parasite fecundity with PZQ treatment. We thereby demonstrated that density-dependence in schistosome populations may complicate evaluation and monitoring of control programmes.

**Electronic supplementary material:**

The online version of this article (10.1186/s13071-017-2533-6) contains supplementary material, which is available to authorized users.

## Background

Neglected tropical diseases (NTDs) are caused by a group of infectious agents that, almost exclusively, affect the rural poor in developing countries [1]. Schistosomiasis, caused by parasitic flatworms, is a disease of profound medical and veterinary importance, affecting over 240 million people, with over 90% of these in sub-Saharan Africa (SSA) [2]. National successes in the control of this important disease have been realised through mass drug administration (MDA) of affected communities, using the current drug of choice, praziquantel (PZQ) [3]. For example, the Schistosomiasis Control Initiative, established in 2002, has assisted Ministries of Health across SSA to deliver more than 250 million PZQ treatments to date. These control programmes, termed preventative chemotherapy (PC), have had impressive effects on reducing schistosomiasis prevalence, infection intensity and associated morbidity [4]. However, only 21% of people estimated to need PZQ received it in 2014 [5]. Commitments such as the development of a World Health Organization (WHO) Roadmap for NTD implementation [6], the inclusion of NTD control in the sustainable development goals and large scale PZQ drug donations and in particular the promise by Merck KGaA to donate 250 million PZQ tablets per year from 2016 [3], have all aided the continued scaling up of such programmes, with the promise of an agenda for elimination of schistosomiasis as a public health problem in some areas [6]. The implementation of such large-scale preventative chemotherapy, however, is likely to be exerting strong and prolonged evolutionary selection pressure on the parasites [7]. These may cause selection towards reduced drug efficacy, but also potentially changes in life history trade-offs affecting, for example, host use, transmission rates and/or parasite virulence. Such selective pressures will increase as the fraction of the parasite population undergoing chemotherapy increases and *refugia* decrease [7].

Clinical efficacy of PZQ is measured as the egg reduction or cure rates and recent data around Lake Victoria have raised concerns that there may be a real possibility of reduced efficacy in communities with a more intensive history of PZQ treatment [8, 9]. However, variations in PZQ efficacy depend on both host and parasite factors, and in the absence of individual level longitudinal data before and immediately after PZQ treatment over several years, or baseline data from before PC, it is difficult to know whether heterogeneity in treatment response is static or changing over time, as would be expected if PZQ resistance was developing. Monitoring of drug efficacy over time in treatment programmes is complicated since ethical considerations mean that if there is testing for, and detection of, parasites immediately following treatment, further treatment is necessary, and thus selected sites will not mimic the treatment history of the rest of the control programme. Moreover, several features of schistosome epidemiology, together with the lack of sensitivity of current diagnostic tools [10], means monitoring the impact of treatment on parasitological indicators is itself complex, not least because it relies on indirect measures of egg output, rather than adult worm burdens. For example, density-dependence is a common feature of macroparasite life-cycles that can influence parasite fecundity, survival and establishment and can act at multiple points of the macroparasite life-cycle. At high parasite population densities, negative density dependence tends to restrict population growth and contribute to the stability of these populations. Interventions that lead to a reduction in parasite populations could thereby be predicted to cause a relaxation of density-dependent restrictions, increasing *per-capita* rates of reproduction or survival, thereby potentially contributing to population persistence and resilience, and complicating the monitoring of chemotherapy success [11].

A key problem in monitoring for PZQ resistance is that though such resistance can be selected for in the laboratory, its molecular basis has not yet been ascertained, and thus genotypic markers for PZQ resistance are currently not available. However, at the individual level, in vitro phenotypic monitoring of larval behaviour when exposed to PZQ has been developed to monitor the phenotypic status of individual parasites, with in vitro PZQ susceptibility correlating to in vivo treatment success [12]. In addition, genetic monitoring using neutral markers such as microsatellites can identify clusters of genetically related parasites indicative of population level changes in parasites [13]. Thus, monitoring the impact of preventative chemotherapy on parasite population genetics, and in particular associations with PZQ susceptibility phenotypes, may be of considerable importance to the continued success of control programmes. Moreover, population genetic studies performed on the accessible miracidial larval stage, enable estimation of adult worm burden and egg per worm output using parentage analysis, which cannot be measured by parasitological field methods alone. The precise nature of negative fecundity density dependence in natural human schistosome populations is not well understood, since the only data that exists is from a single autopsy study [14], and there are no data on the response of this relationship to preventative chemotherapy.

Our previous work, on the *Schistosoma mansoni* parasite population genetics in two schools in the Lake Victoria region in Tanzania [13], was one of the first studies to make use of then recently developed techniques for parasite collection, storage and DNA amplification of schistosome miracidia allowing large numbers of individual miracidia to be analysed directly from stools from infected children [15]. Thus this represented one of the earliest baseline field collections of schistosome miracidia before widespread control of schistosomiasis in sub-Saharan Africa began. After collection of baseline data in April 2005, Bukindo and Kisorya Primary schools received school-based mass treatment of PZQ in June of the same year as part of the first treatment of the National Schistosomiasis Control Programme, and parasite samples were collected from children at the same schools six months later. Despite the large parasite *refugia* (in snails, untreated mammals and untreated people), we demonstrated a significant decrease of allelic richness and heterozygosity of the parasites after only one round of PC including in miracidia isolated from untreated children [13]. Such a community-wide effect on untreated children was interpreted as either a protective immune effect induced by the mass treatment of the population, or more worryingly evidence of selection for potentially drug-resistant parasites. An alternative explanation is that the changes observed were not due to the treatment itself, but to other factors, such as environmental differences between the years of the study, or a combination of both. To date, none of these hypotheses have been confirmed in the natural environment in Tanzania, and studies in other places have reported varying effects of PZQ infection on *S. mansoni* ranging from no evidence of genetic change over time (e.g. in Senegal [16], or Kenya [17]) to clear effects of decreasing diversity and effective population size in response to treatment (e.g. in Brazil, [18]).

The aim of this study was to return to Bukindo and Kisorya Primary schools to measure the effect of five years of the National Schistosomiasis Control Programme Tanzania on the phenotype and genotype of the *S. mansoni* parasite population. Using the same experimental set-up as the baseline study, we compared genetic differentiation and indices of genetic diversity in 2010 samples with baseline samples collected in 2005 and 2006. We performed in vitro tests of PZQ sensitivity and, for the first time in a natural population, compared the genetic profiles of individual miracidia with low or high susceptibility to PZQ in vitro, to quantify genetic differentiation between them. Finally, we assessed evidence of the existence of density dependence in schistosome populations using genetic parentage analysis.

We predicted that: (i) the genetic composition of parasite populations may change over time and in response to PZQ treatment; (ii) in vitro PZQ testing can identify individual parasites with reduced sensitivity to PZQ and that population genetics can ascertain if those with reduced sensitivity are closely related to each other; (iii) the genetic diversity of parasite populations will further reduce with additional PZQ treatment; and (iv) adult worm burdens are non-linearly related to egg output and that this relationship may be affected by PZQ treatment.

## Methods

### Study area, children surveyed and parasite samples collected

As reported elsewhere [13], *S. mansoni* miracidia were collected from children aged 7–11 years old at two schools in close proximity to Lake Victoria, Tanzania, in April 2005, six months prior to the first National Schistosomiasis Control Programme mass school based treatment with PZQ, and again one year later in April 2006. In 2005, miracidia were collected from 38 children at Bukindo Primary School (Ukewere District, Ukewere Island, Mwanza Region) and 42 children from Kisorya Primary School (Bunda District, Mainland, Mara Region), and in 2006 from 18 children at Bukindo Primary School and 29 children from Kisorya Primary School (Additional file 1: Figure S1). The straight-line geographical distance between the villages was approximately 18 km. Bukindo Primary School received PC with PZQ in 2005, 2006, 2007 and 2009, while children from Kisorya Primary School were treated with PZQ in 2005 and 2007 only due to funding restrictions. The two schools were re-visited in July 2010 and at this time the study was explained to the children and they were assured that participation was voluntary. Sixty randomly selected children at each school who provided oral consent were assayed for *S. mansoni* infection by Kato-Katz microscopy over three days (see below). Following data and sample collection, all children in both schools (including those who took place in the survey, any children who had been asked but did not want to take part and all other children attending school) were treated for schistosomiasis with PZQ (40 mg/kg) and soil-transmitted helminths with albendazole (400 mg).

### Determination of infection intensity

In order to determine the prevalence and intensity of schistosome infection, duplicate Kato-Katz thick smears were prepared from three individual stool samples, over three consecutive days, from each child. The infection intensity was expressed as eggs per gram stool (epg) and averaged over the two slides for each day, and then over the three days of collection. All infection data were collected pre-treatment at each time point and there was no direct measure of drug efficacy in terms of egg reduction rate or cure rate so that the selected populations had the same treatment history as the rest of the control programme.

### Collection of *S. mansoni* miracidia from faecal samples

*Schistosoma mansoni* eggs were purified from individual stool samples of all Kato-Katz-positive children as previously described [13, 15] and hatched to obtain individual miracidia, which were stored on Whatman FTA cards® (Whatman International Ltd., Maidstone, UK) until required for genetic analysis.

### In vitro PZQ testing of miracidia

Samples from five children, which yielded sufficient viable miracidia, were used to screen for susceptibility to PZQ using an in vitro phenotypic test based on the change in shape that occurs in susceptible *S. mansoni* miracidia when they are exposed to PZQ. In brief, susceptible miracidia first become tadpole and then dumbbell shaped upon PZQ exposure, with the degree of shape change and activity level correlated to drug susceptibility and in vivo drug efficacy [12]. For each child, between 40 and 60 individual miracidia were collected in 18 μl water. Using a microscope the wildtype phenotype at baseline was confirmed for each individual miracidium. Following that, 2 μl of a 5 × 10^−6^ M PZQ solution (Sigma-Aldrich, Gillingham, UK) were added, in order to expose every miracidium to a final PZQ concentration of 1 × 10^−6^ M in 20 μl total volume. After 5 min incubation at room temperature, resistance levels of individual miracidia were microscopically classified according to observed phenotypical changes: “less susceptible” - normal shape following PZQ exposure, “intermediate susceptibility/resistance” - tadpole shape, “fully susceptible” - dumbbell shape [12], and stored on FTA Whatman cards as described above.

### Microsatellite analysis of individual miracidia

Molecular analysis was carried out on up to 20 miracidia per child from the 2010 collections depending on the infection intensity of the child. We replicated the identical methodologies of the original 2005 and 2006 surveys in order that the data were directly comparable [13], since the original miracidial samples were no longer available for re-analysis. In brief, DNA preparation was carried out on the Whatman cards as per the manufacturers protocol (Whatman FTA cards®). PCR was carried out using the previously published multiplex assay using seven pairs of microsatellite primers [15], namely SMD28, SMDA28, SDM25, SMD89, CA11-1, SMS9-1 and SMU31768. Forward primers were labelled using 6-FAM, PET, VIC and NED dyes. Microsatellite sizing was performed on the same ABI Prism 3730 Genetic Analyser with a LIZ-500 size standard (Applied Biosystems, Cheshire, UK) as the 2005 and 2006 samples.

### Statistical analyses

#### Prevalence and infection intensity of schistosomiasis in 2005–2010

Estimates of prevalence and infection intensity were calculated using data from the 2010 survey of approximately 60 children at each school, and were compared with estimates of prevalence and infection intensity from 2005 and 2006. These were based on larger surveys of approximately 200 children per school undertaken as part of the National Schistosomiasis Control programme monitoring and evaluation (Schistosomiasis Control Initiative), unpublished data [13]. To compare the infection intensity at the two schools in 2010 we used a two-sample t-test with equal variances of logarithmically-transformed data.

#### Population genetic analyses

Allele sizes were calculated in Genemapper (v4) and manually checked using the same bin sizes as for the 2005 and 2006 samples. Subsequent analysis was restricted to miracidia with at least 4 allele calls. There was an average of 7% missing data present per locus but no evidence that there were systematic differences between the years in the amount of missing data (*χ* ^2^ = 1.04, *df* = 2, *P* = 0.41). All population genetic measures were calculated at an infrapopulation level (where all the parasites within a host are considered as a single infrapopulation), except where explicitly stated.

#### Parentage analysis, estimates of adult worm burden, and reproductive success of adult worms

The estimation of full-relationships between the miracidia of individual children was carried out separately using Colony version 2.0.6.1 [19]. Colony implements a maximum likelihood algorithm when comparing different sibship configurations and also allows for genotyping error [20]. Genotyping error was calculated from repeat amplifications of 30 adult worms (from which multiple DNA samples could be obtained) and had a mean value of 3.6% (range 0–7%). The definition of genotyping error included alleles which did not amplify (missing data). Colony allows for a number of assumptions regarding mating behaviour of parental worms, and we assumed that adult worms were monogamous, but that clonal worms could be present, as suggested by recent analysis of the mating behaviour of schistosome system [21]. Sibship relationships are then used to estimate the number of unique adult worm genotypes present. To investigate the presence of density dependent fecundity, the worm burdens of individual children estimated using Colony, together with their individual infection intensity, were used to estimate the parasite reproductive success, measured as the mean number of eggs per worm pair.

Differences between parasite within-child infrapopulations collected at Bukindo and Kisorya schools in 2005, 2006 and 2010, in terms of their mean adult worm burden and mean parasite reproductive rate, were investigated using linear regression modelling in Stata Statistical Software (StataCorp LP, USA), incorporating an interaction term to investigate whether differences between years were similar at the two schools under study. Child age, sex, and miracidial sample size were used as covariates and survey commands were used to account for within-school clustering.

#### Diversity of infections

Summary statistics for expected heterozygosity (He), observed heterozygosity (Ho) and allelic richness (Ar) across each population (each child at each time point) were created in FSTAT.2.9.3.2 [22]. Allelic richness is suitable for comparison of samples of different sizes since it rarefies to the smallest sample size in the dataset, and is thus particularly useful for schistosome population genetics where the number of miracidia collected is in part dependent on infection intensity. The alternative approach of excluding small samples will bias the dataset against lighter intensity infections, which might be particularly common following successful PZQ treatment. Differences in He, Ho and Ar per within-child infrapopulation between 2005, 2006 and 2010 at each of the two schools, were tested for using linear regression in Stata Statistical Software (StataCorp LP, USA) using child age, sex, school and miracidial samples size as covariates and using survey commands to account for within-school clustering. An interaction term was included to assess whether differences between years was similar at the two schools, but was excluded from the final model if non-significant. Differences in summary statistics between the parasite infrapopulations at each of the three time points were compared using a non-parametric permutation model in FSTAT with 5000 permutations.

#### Population structure

As a measure of genetic distance between the parasite populations of individual children, a matrix of Cavalli-Sforza and Edwards’ chord distances [23] was estimated using Powermarker [24] and visualised using a minimum spanning network in Poppr [25] and a neighbour joining clustering algorithm (NJ). The reliability of NJ phenograms was assessed by bootstrapping over loci with 100 replications using CONSENSE [26]. Two separate analyses were carried out, one comparing the parasite populations of all individual children at each school and time point, and the second restricted to those samples in 2010 which had been assessed for in vitro PZQ susceptibility.

Hierarchical Wrights’ FST statistics measuring evidence of genetic differentiation between years, and between children within years were calculated for each school separately using Hierfstat [27]. *P*-values were calculated by 10,000 random permutations. A hierarchical analysis is the most robust analysis as it accounts for the potential relatedness of miracidia within individual children, which will tend to inflate estimates of classical Wrights’ FST statistics [28] but separate analyses were necessary for Bukindo and Kisorya school due to the design of the study that meant that school and year of collection were crossed rather than nested factors [29]. In order to investigate the relative importance of time versus individual level and between school variation, pairwise FST statistics were calculated between all of the parasite infrapopulations and an average calculated for each school/time point combination. Pairwise FST statistics were also calculated between the 2005, 2006 and 2010 component populations (where all the miracidia at each time point and school were pooled) for each school.

## Results

### Prevalence and infection intensity of schistosomiasis in 2005–2010

Prevalence and infection intensity of *S. mansoni* in children aged 7 to 11 years is shown in Table 1. In 2010, Bukindo and Kisorya Primary Schools had comparable prevalences of 95 and 93%, respectively, but infection intensities differed significantly with a mean 111 epg at Bukindo Primary School, and 444 epg at Kisorya Primary School (*t*_(65)_ = -4.04, *P* = 0.001). Combining these 2010 data with previously published baseline data (2005) and data six months after the first round of PC (2006) [13], we observed that, although different between the schools, infection intensities remained stable over time within both of the study sites (Table 1), Kisorya Primary School, which had received two mass treatments and Bukindo Primary School, which had received four treatments in the time period. We note that these represent pre-treatment infection levels, and do not directly measure drug efficacy since re-infection may have occurred between survey points.

**Table 1:** Prevalence (± 95% confidence interval, CI) and mean arithmetic infection intensity (± 95% CI) of *Schistosoma mansoni* at two schools in Lake Victoria region of Tanzania in April 2005, April 2006 and July 2010

School	Year	Prevalence (%) (95% CI)	Mean infection intensity (epg) (95% CI)	No. of children surveyed (*n*)
Bukindo	2005	60.3 (56.6–64.1)	134.5 (114.2–154.5)	174
Bukindo	2006	77.0 (73.4–80.3)	65.5 (49.9–81.1)	160
Bukindo	2010	95.5 (88.6–98.0)	116.2 (81.2–151.2)	66
Kisorya	2005	90.4 (88.4–92.3)	421.9 (349.8–494.0)	228
Kisorya	2006	93.0 (91.2–94.8)	427.4 (352.0–502.8)	205
Kisorya	2010	92.7 (88.6–96.7)	444.4 (284.6–604.2)	41

Bukindo School received treatment in November 2005, 2007, 2008 and 2009 and Kisorya School received treatment in November 2005 and 2007 only

### In vitro drug-susceptibility testing of miracidia

Following screening in both schools, children with medium (100–399 epg) or high (>400 epg) infection intensities, and stool samples which yielded sufficient numbers of viable miracidia within 20 min, were selected for in vitro PZQ susceptibility testing. In 2010, five children complied with these criteria and 35–65 miracidia per child were tested being exposed to 1 × 10^−6^ M PZQ. As shown in Table 2, all five children hosted worms whose offspring contained miracidia that showed reduced in vitro susceptibility to PZQ. A total of 234 miracidia were screened and, on average, 6% were less susceptible and retained a normal shape following in vitro exposure to PZQ. The remaining miracidia all showed clear phenotypic changes that indicated PZQ-susceptibility, becoming tadpole shaped, however, no dumbbell shapes (the more extreme shape change associated with greater PZQ susceptibility) were observed in any samples tested.

**Table 2:** In vitro praziquantel-susceptibility of individual *S.mansoni* miracidia isolated from 5 children in the Lake Victoria region of Tanzania in July 2010, as measured by five minute exposure to 1 × 10^−6^ M PZQ and classified microscopically by shape as normal (indicating reduced susceptibility to PZQ) or tadpole shape (sensitive to PZQ)

School	Child ID	PT^a^	Individual infection intensity (epg)	Total no. of miracidia tested (*n*)	No. of less susceptible miracidia (shape normal)	No. of susceptible miracidia (shape tadpole)	Percentage of less susceptible miracidia (95% CI)
Bukindo	1026	+	400	64	6	58	9 (2.0–16.0)
Kisorya	0012	–	1216	36	1	35	3 (0–8.6)
	0017	–	2520	41	2	39	5 (0–11.7)
	1002	+	330	45	4	42	7 (0–14.5)
	1010	+	768	48	2	46	4 (0–9.5)
Total	–		–	234	15	220	5.6 (3.0–9.0)

PT indicates whether children had a previous history of PZQ treatment

^a^+, yes; −, no

### Longitudinal genetic analysis of *S. mansoni* population 2005–2010

In 2010, miracidia were successfully stored from nine children at Bukindo Primary School and from 18 children at Kisorya Primary School. For the longitudinal analysis, we analysed up to 20 miracidia per child depending on availability and thus analysed 107 miracidia from Bukindo Primary School and 245 miracidia from Kisorya Primary School in 2010, with a school treatment history of four and two rounds of PC, respectively, and compared them with previously collected miracidia from the same schools in 2005 (baseline) and 2006 (6 months after first PC), to give a total dataset of 1936 miracidia. The mean number of miracidia per child (±95% confidence interval) in 2010 was 13.0 ± 0.70 which was not significantly different from the mean number of miracidia in 2005 (12.2 ± 0.63) or 2006 (13.8 ± 0.56) (*F*_(2,148)_ = 1.66, *P* = 0.194). One hundred and thirty miracidia, which had been characterised as PZQ-susceptible or not by the in vitro phenotypic assay, were analysed separately.

### Parentage analysis, estimates of adult worm burden, and reproductive success of adult worms

**Fig. 1:**
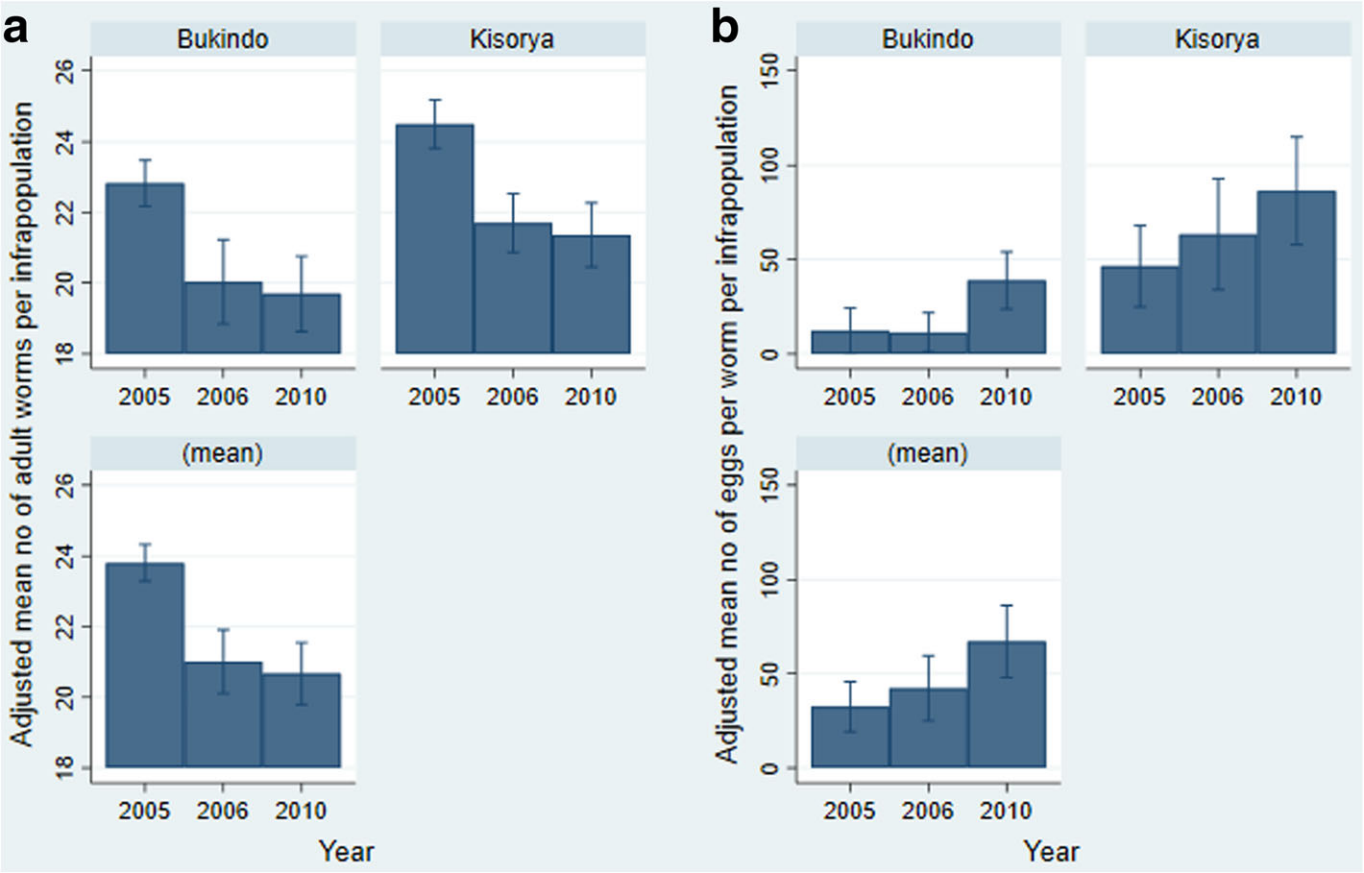
Mean adult worm burden (**a**) and eggs per adult female worm (**b**) for the parasite infrapopulations of individual children, adjusted for child age and sex and miracidial sample size, at two schools near Lake Victoria in 2005, 2006 and 2010

Adult worm burdens were estimated from genetic data using parentage analysis. As shown in Fig. 1a, there was a reduction in the estimated adult worm burden over time and regression modelling confirmed that this was a significant difference in both 2006 and 2010 compared to the baseline collection (2006: *β* = -5.39, *t* = -5.80, *P* < 0.001; 2010: *β* = -2.46, *t* = -3.82, *P* < 0.001). The number of miracidia was significantly associated with the estimated number of worms (*β* = 1.56, *t* = 41.5, *P* < 0.001) but there was no significant association of child age (*t* = 0.67, *P* = 0.50) or sex (*t* = 0.86, *P* = 0.39) with the estimated number of adult worms in this dataset and the fitted model explained 92% of the variation in the estimated number of adult worms. The mean number of eggs per adult worm pair was higher in parasite infrapopulations in 2010 than 2006 and 2005 (*β* = 34.3, *t* = 2.64, *P* = 0.009), and as shown in Fig. 1b, and was higher at Kisorya than at Bukindo school (*β* = 41.4, *t* = 4.68, *P* < 0.001).

### Diversity of infections

**Fig. 2:**
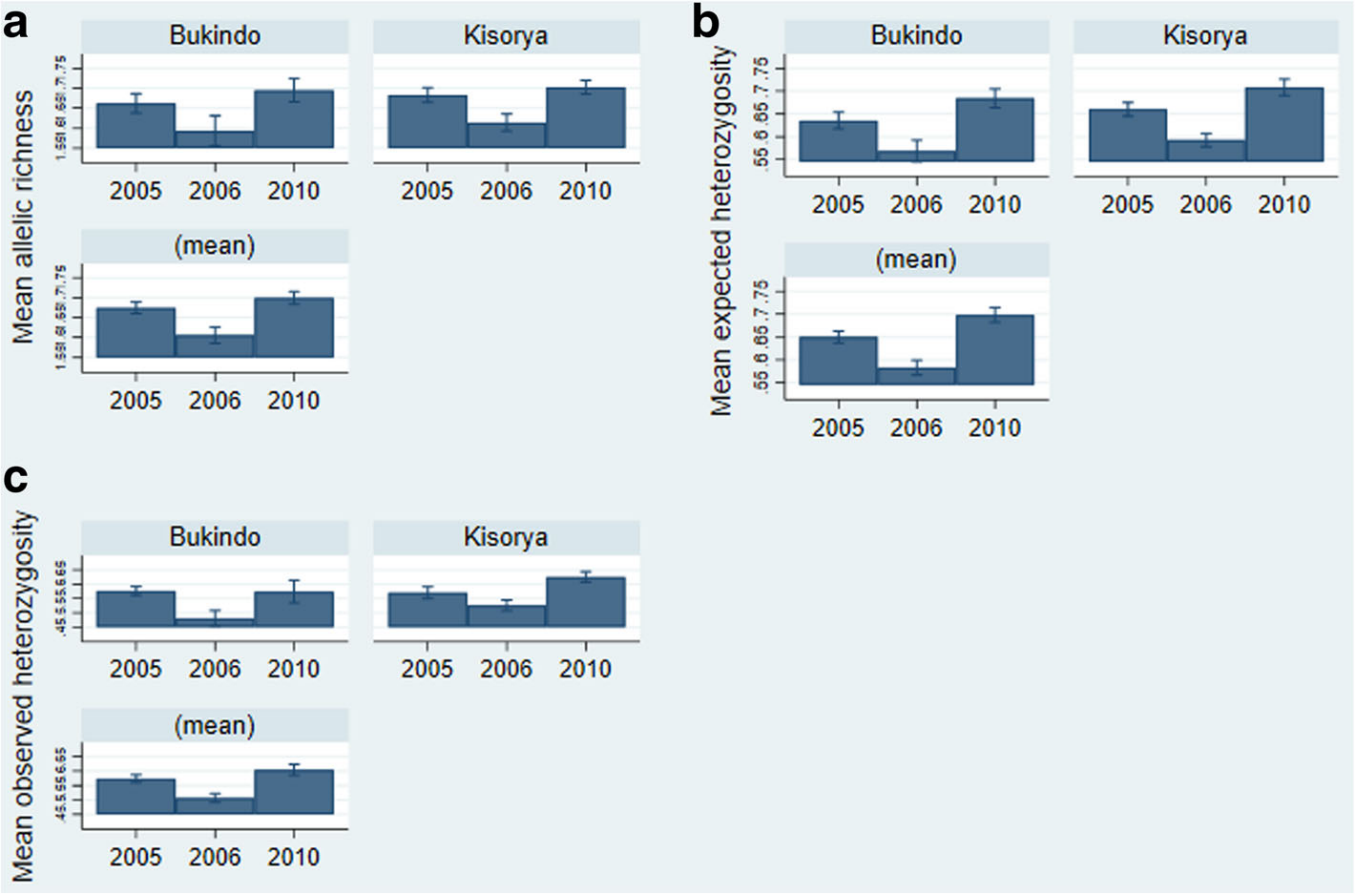
Mean (± 95% confidence intervals) allelic richness (**a**) and expected (**b**) and observed heterozygosity (**c**) for the parasite infrapopulations of individual children, adjusted for child age and sex and miracidial sample size, at two schools near Lake Victoria in 2005, 2006 and 2010

There was a significant difference in genetic diversity between parasite populations collected in 2006 and 2005, and between those collected in 2010 and 2005. As previously reported, and shown in Fig. 2a, there was a reduction in allelic richness between 2006 and the baseline 2005 collection (*β* = -0.067, *t* = 5.32, *P* < 0.001). This was not, however, maintained in 2010 and genetic diversity was slightly higher in the 2010 collections relative to the baseline collection (*β* = 0.024, *t* = 2.09, *P* = 0.04). There was no difference in allelic richness between the schools (*β* = 0.018, *t* = 1.75, *P* = 0.09). As shown in Fig. 2b, c, similar patterns were seen in both expected and observed heterozygosity (He 2006 compared to 2005: *β* = -0.07, *t* = -6.46, *P* < 0.001; 2010 compared to 2005: *β* = 0.05, *t* = 4.46, *P* < 0.001; Ho 2006 compared to 2005: *β* = -0.07, *t* = 6.62, *P* < 0.001; 2010 compared to 2005: *β* = 0.03, *t* = 2.62, *P* = 0.01), although there was a significant lower diversity at Bukindo school (He: *β* = 0.024, *t* = 2.37, *P* = 0.02; Ho: *β* = 0.19, *t* = 2.09, *P* = 0.04), which might reflect differences in infection intensities between the two schools, despite statistical attempts to correct for the miracidial sample size. Observed heterozygosity was lower than expected heterozygosity in all populations, although there was no evidence of change in this relationship over time. The significant differences between years were confirmed by non-parametric permutation tests (Ar *P* < 0.001, Ho *P* < 0.001, He *P* < 0.001).

### Population structure

**Fig. 3:**
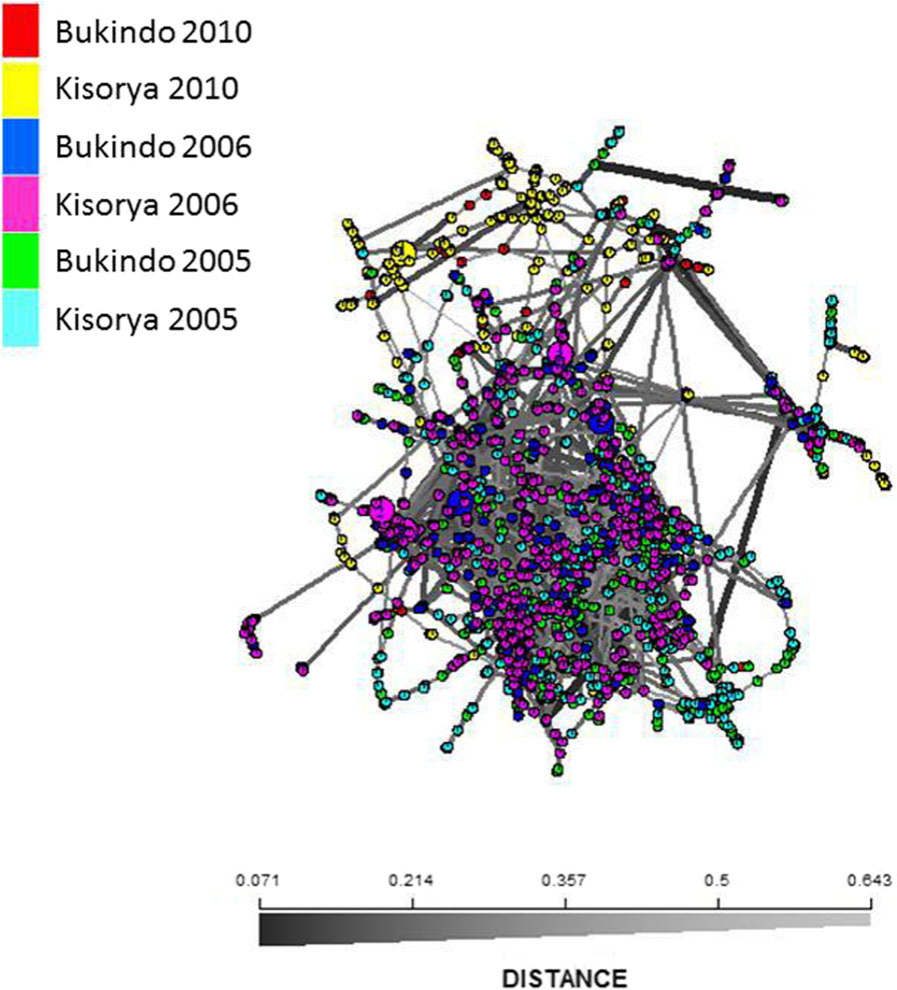
Minimum spanning distance calculated using Edwards Cavelli-Chord distance between parasite infrapopulations collected in two schools (Bukindo Primary School, Ukewere Island and Kisorya Primary School, Mara District) in 2005, 2006 and 2010

The genetic similarity of parasite infrapopulations collected in the two schools at each time point are shown in Fig. 3. There was clear clustering by year of collection, with the later collections of 2010, in particular clustering together. However, there was considerable overlap between infrapopulations collected at the different schools particularly in the earlier two time points. This was confirmed by hierarchical FST statistic analysis, which indicated that most of the variation was observed within individual children but that there was significant more variation between the parasite populations of children sampled in different years than within years at both Bukindo and Kisorya schools. At Bukindo school, FST (+ 95% confidence limits) between years was 0.043 (0.025–0.057), and within years 0.020 (0.013–0.036), while FIS was estimated as 0.26 (0.16–0.41). At Kisorya school a similar pattern of between years FST 0.052 (95% CI: 0.030–0.074), 0.013 (0.006–0.027) between children within years and 0.29 (0.20–0.40) between miracidia within children (FIS) was observed. Analysis of pairwise FST statistics (Table 3), likewise, demonstrated a larger differentiation between years, than between schools within years, measured both as the component populations and as the mean of the pairwise FST statistics for the within-child infrapopulations.

**Table 3:** Pairwise FST between *Schistosoma mansoni* component populations and within-child *Schistosoma mansoni* infrapopulations at two schools in Lake Victoria region of Tanzania in April 2005, April 2006 and July 2010

	Bukindo 2005	Bukindo 2006	Bukindo 2010	Kisorya 2005	Kisorya 2006	Kisorya 2010
Pairwise FST between schools and years (component populations)						
Bukindo 2005	na					
Bukindo 2006	0.023	na				
Bukindo 2010	0.064	0.096	na			
Kisorya 2005	0.004	0.023	0.061	na		
Kisorya 2006	0.023	0.022	0.095	0.017	na	
Kisorya 2010	0.069	0.064	0.003	0.064	0.098	na
Mean of between infrapopulation FST						
Bukindo 2005	0.031					
Bukindo 2006	0.047	0.028				
Bukindo 2010	0.076	0.109	0.012			
Kisorya 2005	0.026	0.044	0.075	0.024		
Kisorya 2006	0.039	0.019	0.098	0.034	0.012	
Kisorya 2010	0.082	0.113	0.012	0.077	0.102	0.007
Overall mean between infrapopulation FST		0.048				

### Phylogenetic analysis of miracidia with differing PZQ-susceptibilities

**Fig. 4:**
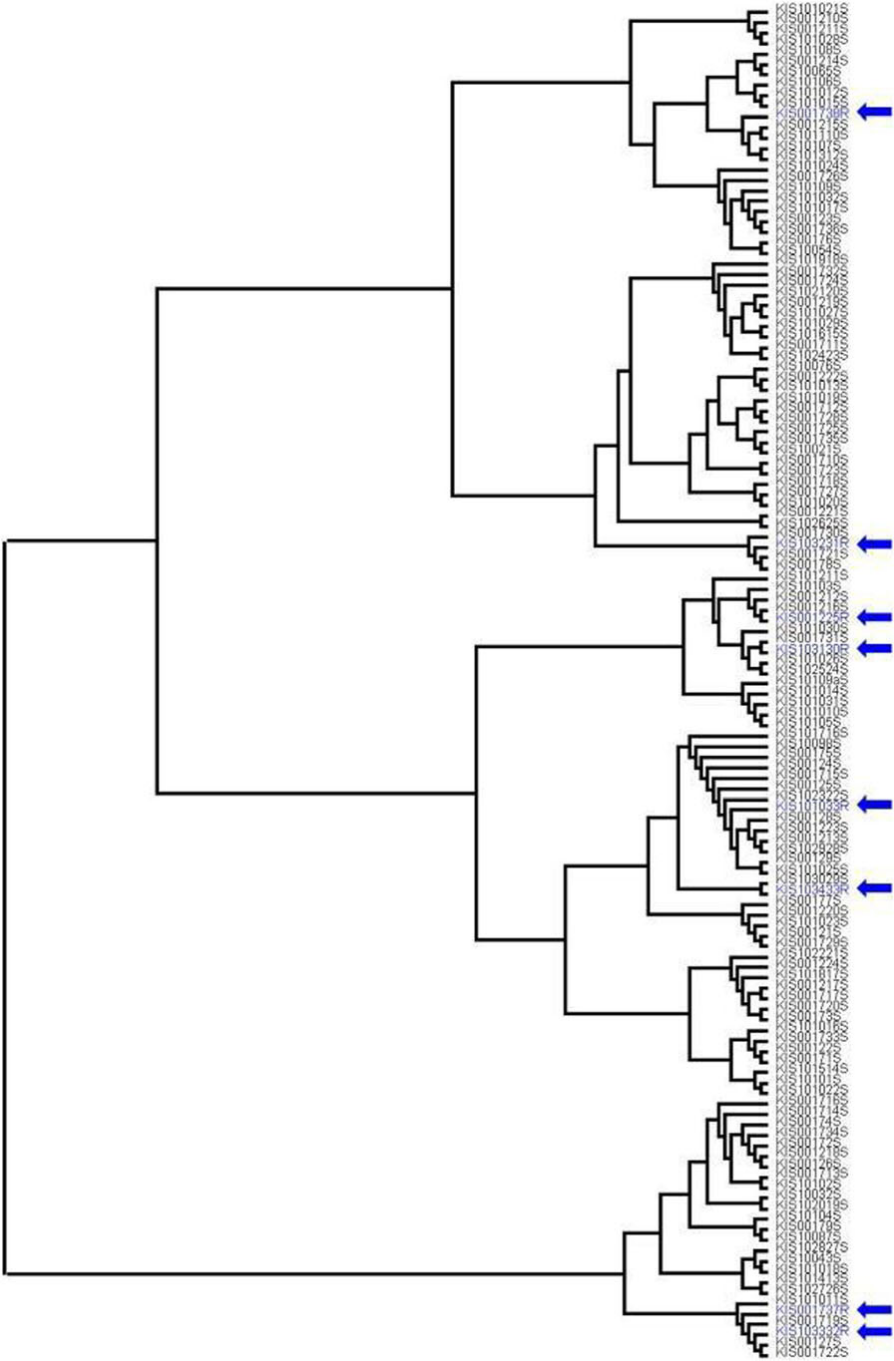
Genetic similarity of individual parasites subject to in vitro parasite testing using Edwards Cavelli-Chord distance and visualised using a neighbour-joining (NJ) phenogram. A consensus tree from 100 bootstrap repetitions is shown. Arrows show the presence of individuals with reduced susceptibility to PZQ in vitro

One hundred and twenty one susceptible and eight less susceptible miracidia isolated from Kisorya Primary School were successfully genotyped. There was no evidence that less susceptible miracidia identified by in vitro PZQ testing were related and therefore might constitute a genetic cluster, as demonstrated using minimum spanning network analysis and a consensus NJ tree between individuals (Fig. 4).

## Discussion

In host populations that are subject to frequent MDA, the parasite population may be predicted to respond with genetic changes. In the case of PZQ, the only drug currently in distribution for schistosomiasis, the emergence of *S. mansoni* strains that have lost susceptibility is a major concern. With the current lack of specific genetic markers for PZQ resistance [7], we rely on in vivo and in vitro efficacy studies [8, 12] and population genetics using neutral markers to understand parasite population response to the selective pressures of wide-scale drug treatment [13]. Here, for the first time, we combine results of in vitro phenotypic monitoring with genotypic data to understand the effect of five years of a national control programme on *S. mansoni* parasite populations. Our results provide evidence of the presence of parasites with reduced susceptibility to PZQ in the parasite population five years after the start of a National Control Programme using PC with PZQ (and the anti-nematode albendazole), as evidenced by an in vitro phenotypic assay based on larval response to PZQ. However, there was no evidence that parasites showing phenotypically reduced susceptibility clustered together genetically, as would be expected following an ongoing selective sweep. Genetic data were also used to estimate adult worm burden, demonstrating a reduction of worm load over time, despite little evidence of long term reduction in parasite infection intensity. Genetic diversity of infections did not reduce further over time but there was evidence of change in the parasite populations in their genetic composition.

Prevalence and infection intensity of schistosomiasis remained high at both study sites, although infection intensity was significantly lower at Bukindo School on Ukewere Island, compared to the mainland school, Kisorya. Both schools received school-based PC in 2005 and 2007 under the National Control Programme, but Bukindo additionally received community-wide PC for all individuals over five years old in 2008 and 2009. Drug efficacy in terms of egg reduction or cure rate immediately after treatment was not measured, such that these schools had the same treatment history as the wider population undergoing schistosomiasis control. The temporal trends in the prevalence and intensity data suggest that the PC treatments were not having a major impact on the longer-term prevalence and intensity, possibly due to rapid re-infection in this high transmission area. It should be noted, however, that there is wide heterogeneity in individuals’ schistosomiasis infection levels, and this study was not powered to detect changes in prevalence and infection intensity following MDA treatment, for which much larger samples sizes are required (see e.g. [30]). Indeed, a key finding of this study is that monitoring of infection intensity using egg output may have additional problems above the widely reported low sensitivity of Kato-Katz [10], namely that we report non-linear relationships between adult worm burden and egg output, that may also be influenced by treatment history. We used parentage analysis to estimate the adult worm burden from offspring genetic data, a technique widely used to estimate population sizes in conservation science, but with more restricted use in parasite biology ([31], but see, e.g. [32, 33]). Our results suggest that despite no evidence of a reduction in mean infection intensity, adult worm populations may indeed be declining, masked by the fact that their egg output per worm pair may be increasing, resulting in a lack of significant change in mean infection intensity and therefore completely undetected by the standard Kato-Katz techniques. The reduced worm numbers resulted in a higher average parasite reproductive success (measured as mean egg output per adult worm pair) for parasite populations following MDA, and could indicate the relaxation of density-dependent constraints following successful killing of adult worms by PZQ. Density dependence may thereby potentially be contributing to population persistence and resilience, and complicating the monitoring of chemotherapy success. Demonstration of the existence of density-dependence in natural schistosome populations is difficult due to non-accessibility of adult worms due to their location in the blood vessels of the mesenteric system around the intestine (*S. mansoni*) or bladder (*S. haematobium)* and the only data that currently exists for human populations is from a single autopsy study [14], thus population genetic data and parentage analysis is a useful addition to this field. A drawback is evidently that worms are not directly observed, and thus the reliability of the data is hard to prove, being dependent on the reliability of algorithms for inferring parentage. This is a particular challenge in schistosomiasis where asexual reproduction in the intermediate snail host may result in the existence of clonal adult worms within individual human hosts. However, there is little evidence that the proportion and/or distribution of clonal adult worms would change over time, therefore supporting our interpretations. In particular, we did not see any changes in the relationship of observed and expected heterozygosity which might reflect changes in parasite mating behaviour, indicative of an increase in the presence of clonal adult worms. The results of this study, however, do suggest that further investigation of parentage techniques, perhaps using more molecular markers, and the consideration of density-dependent factors in mathematical modelling of treatment impact [11] is warranted. Such data will be of particular importance should drug resistance arise, as parasites surviving treatment, will have the density-dependent pressures reduced and the surviving more resistant strains may rapidly increase in their proportional contribution to the gene pool.

Clustering, hierarchical FST and pairwise FST analyses confirmed that parasite populations had changed over the five-year time period since the baseline collections. Re-analysis confirmed that a reduction in genetic diversity was seen between the first and second collections, but later collections showed no further reduction in diversity. One concern from the original study was that a reduction in parasite diversity may suggest the selection of parasite sub-populations able to survive MDA treatment. Thus a further aim of this study was to consider whether we were able to detect strains that are less susceptible to PZQ. We used an in vitro larval behaviour assay, which is positively correlated with in vivo treatment success [12]. We demonstrated that 6% of miracidia were of “resistant or less susceptible”, normal phenotype despite in vitro drug exposure, but 94% had a contracted shape and therefore were susceptible to PZQ. A similar percentage was also shown in the Ugandan study [12], and this correlates well with the approximate fraction of people that did not respond at a population level in more recent studies in Uganda who had previously received 1–5 PZQ treatments [8]. Although a few “resistant” miracidia were detected, one would still classify the overall schistosome population as susceptible, since Liang and colleagues determined that PZQ-sensitive worm strains from Puerto Rico, Kenya and Egypt produced 67–100% of miracidia which responded to in vitro PZQ exposure with phenotypic changes [34]. Indeed, using population genetic analysis, we investigated whether the in vitro identified less susceptible miracidia clustered into a group of genetically related miracidia. However, no clustering was observed using the neutral markers in this study, suggesting that these parasites were not closely genetically related. The lack of relatedness argues against a selective sweep of a small number of “resistant” strains in this population, but rather supports the existence of a significant minority of independently evolving populations with an existing low susceptibility to PZQ. Therefore, we do not have evidence of an observed impact of PZQ treatment on the selection of resistant phenotypes in this study area. However, despite these promising findings, we did have some cause for concern. Although in vitro testing would classify this Tanzanian *S. mansoni* population as susceptible to PZQ, it was noted that all of the susceptible miracidia responded with a tadpole shape, indicative of intermediate susceptibility, rather than a dumbbell shape associated with full susceptibility, in contrast to samples from Uganda which consisted of equal proportions of tadpole and dumbbell shapes, i.e. a mixture of intermediately and fully susceptible [12]. Moreover, the study by Crellen et al. [8], identified evidence of a reduction in efficacy (about 10% reduction) only in populations that had received 8 or 9 rounds of PC. Due to the key importance of continued PZQ efficacy to the success of these highly important control programmes, which are currently protecting millions of people from severe disease, we suggest that continued long-term monitoring of PZQ efficacy and the genetics of schistosome populations using larger sample sizes, and across wider geographic areas, are required. Although we found no evidence of genetic relatedness and shared identity between individuals with low natural susceptibility to PZQ using neutral markers, comparison of individual miracidia with varying in vitro susceptibility using whole genome sequencing may identify areas of the genome that may be directly related to PZQ action and confirm if single or multiple modes of action are responsible for increasing tolerance [8]. Such studies will also be critical for the identification of molecular markers associated with PZQ resistance and susceptibility and thus allow us to directly monitor the effect of MDA and varying treatment regimes on schistosome populations across the required wide geographic area.

## Conclusions

This study revisited two schools in the Lake Victoria area of Tanzania five years after the introduction of a National Control Programme to conduct follow-up studies of the epidemiology and population genetics of *S. mansoni* populations that had been investigated before treatment began, and six months after, the first school-based PC treatment [13]*.* Using the same experimental set-up as the original study, we identified no further decrease in the intensity or genetic diversity of *S. mansoni* infections within school children but observed density-dependent effects on the worm population following treatment, which would urge caution in interpreting infection intensity results as measured by egg output. Our longitudinal analysis showed further changes in the genetic make-up of schistosome populations which also included miracidia that were less susceptible to PZQ in vitro. However, we found no evidence of genetic similarity between parasites that were less susceptible to PZQ in vitro, as might be expected given a large scale selective sweep and the overall *S. mansoni* population at the study sites in Tanzania’s Lake Victoria region still be classified as susceptible in 2010. We suggest that monitoring of the parasite population and its responsiveness towards PZQ is strongly advisable and that studies on cost-of-resistance [35] in natural schistosome populations should be conducted using parentage analysis to estimate parasite reproductive success and any trade-offs between resistance and other fitness traits since this could potentially help prepare us for the management of potentially emerging PZQ-resistant strains.

## Additional file

Additional file 1: Figure S1. Location of study sites in Tanzania. In order to collect *S. mansoni* miracidia, 7–11 year old children from two schools of Tanzania’s highly endemic Lake Victoria region were sampled. Bukindo Primary School is situated on Ukerewe Island inside Lake Victoria (Ukerewe District, Mwanza Region). The second school studied was Kisorya Primary School (Bunda District, Mara Region) which is located on the mainland. Both schools are in close proximity to the lakeshore. (TIFF 1924 kb)

## Abbreviations

Ar: allelic richness
He: expected heterozygosity
Ho: observed heterozygosity
MDA: mass drug administration
NJ: neighbour-joining
NTD: neglected tropical disease
PC: preventative chemotherapy
PZQ: praziquantel
SSA: sub-Saharan Africa
WHO: World Health Organization

## References

[1] Hotez PJ, Fenwick A, Savioli L, Molyneux DH (2009). Rescuing the bottom billion through control of neglected tropical diseases. Lancet.

[2] Colley DG, Secor WE, King CH (2014). Human schistosomiasis. Lancet.

[3] Webster JP, Molyneux DH, Hotez PJ, Fenwick A (2014). The contribution of mass drug administration to global health: past, present and future. Phils Trans R Soc Lond B.

[4] Fenwick A, Webster JP, Bosque-Oliva E, Blair L, Fleming FM, Zhang Y (2009). The schistosomiasis control initiative (SCI): rationale, development and implementation from 2002–2008. Parasitology.

[5] Schistosomiasis WHO. Number of people treated worldwide in 2014. Weekly Epid Theatr Rec. 2016;91:53–60. 26852429

[6] WHO (2012). Accelerating work to overcome the global impact of neglected tropical diseases -A roadmap for implementation.

[7] Webster JP, Gower CM, Norton AJ (2008). Evolutionary concepts in predicting and evaluating the impact of mass-chemotherapy schistosomiasis control programmes on parasites and their hosts. Evol Apps.

[8] Crellen T, Walker M, Lamberton PH, Kabatereine NB, Tukahebwa EM, Cotton JA, Webster JP (2016). Reduced efficacy of praziquantel against *Schistosoma mansoni* is associated with multiple rounds of mass drug administration. Clin Infect Dis.

[9] Zwang J, Olliaro PL (2014). Clinical efficacy and tolerability of praziquantel for intestinal and urinary schistosomiasis - a meta-analysis of comparative and non-comparative clinical trials. PLoS Negl Trop Dis.

[10] Lamberton PH, Kabatereine NB, Oguttu DW, Fenwick A, Webster JP (2014). Sensitivity and specificity of multiple Kato-Katz thick smears and a circulating cathodic antigen test for *Schistosoma mansoni* diagnosis pre- and post-repeated-praziquantel treatment. PLoS Negl Trop Dis.

[11] Anderson RM, Turner HC, Farrell SH, Truscott JE (2016). Studies of the transmission dynamics, mathematical model development and the control of schistosome parasites by mass drug administration in human communities. Adv Parasitol.

[12] Lamberton PH, Hogan SC, Kabatereine NB, Fenwick A, Webster JP (2010). *In vitro* praziquantel test capable of detecting reduced *in vivo* efficacy in *Schistosoma mansoni* human infections. Am J Trop Med Hyg..

[13] Norton AJ, Gower CM, Lamberton PH, Webster BL, Lwambo NJ, Blair L (2010). Genetic consequences of mass human chemotherapy for *Schistosoma mansoni*: population structure pre- and post-praziquantel treatment in Tanzania. Am J Trop Med Hyg..

[14] Cheever AW (1968). A quantitative post-mortem study of schistosomiasis mansoni in man. Am J Trop Med Hyg.

[15] Gower CM, Shrivastava J, Lamberton PHL, Rollinson D, Emery AM, Webster BL (2007). Development and application of an ethical and epidemiologically appropriate assay for the multi-locus analysis of *Schistosoma mansoni*. Parasitology.

[16] Huyse T, Van den Broeck F, Jombart T, Webster BL, Diaw O, Volckaert FA (2013). Regular treatments of praziquantel do not impact on the genetic make-up of *Schistosoma mansoni* in northern Senegal. Infect Genet Evol.

[17] Lelo AE, Mburu DN, Magoma GN, Mungai BN, Kihara JH, Mwangi IN (2014). No apparent reduction in schistosome burden or genetic diversity following four years of school-based mass drug administration in Mwea, Central Kenya, a heavy transmission area. PLoS Negl Trop Dis.

[18] Barbosa LM, Reis EA, Dos Santos CR, Costa JM, Carmo TM, Aminu PT (2016). Repeated praziquantel treatments remodel the genetic and spatial landscape of schistosomiasis risk and transmission. Int J Parasitol.

[19] Jones OR, Wang J (2010). Colony: a program for parentage and sibship inference from multilocus genotype data. Mol Ecol Resour.

[20] Wang J, Whitlock MC (2003). Estimating effective population size and migration rates from genetic samples over space and time. Genetics.

[21] Steinauer ML (2009). The sex lives of parasites: investigating the mating system and mechanisms of sexual selection of the human pathogen *Schistosoma mansoni*. Int J Parasitol.

[22] Goudet J. FSTAT: a computer program to calculate F statistics. Version 2.9.3.2. https://www2.unil.ch/popgen/softwares/fstat.htm. Acessed Nov 2017.

[23] Cavalli-Sforza LL, Edwards AWF (1967). Phylogenetic analysis: models and estimation procedures. Am J Hum Genet.

[24] Liu J, Muse SV (2005). Powermarker: integrated analysis environment for genetic marker data. Bioinformatics.

[25] Felsenstein J. PHYLIP (Phylogeny Inference Package) version 3.6. Seattle: Department of Genome Sciences, University of Washington. http://evolution.genetics.washington.edu/phylip.html. Accessed Nov 2017.

[26] Kamvar ZN, Tabima JF, Grunwald NJ (2014). Poppr: an R package for genetic analysis of populations with clonal, partially clonal, and/or sexual reproduction. PeerJ.

[27] Goudet J (2005). Hierfstat, a package for R to compute and test variance components and F-statistics. Mol Ecol Notes.

[28] Rudge JW, Carabin H, Balolong EJ, Tallo V, Shrivastava J, Lu DB (2008). Population genetics of *Schistosoma japonicum* within the Philippines suggest high levels of transmission between humans and dogs. PLoS Negl Trop Dis.

[29] de Meeus T, Goudet JA (2007). Step-by-step tutorial to use HierFstat to analyse populations hierarchically structured at multiple levels. Infect Genet Evol.

[30] Koukounari A, Gabrielli AF, Touré S, Bosqué-Oliva E, Zhang Y, Sellin B (2007). *Schistosoma haematobium* infection and morbidity before and after large-scale administration of praziquantel in Burkina Faso. J Infect Dis.

[31] Criscione CD, Poulin R, Blouin MS (2005). Molecular ecology of parasites: elucidating ecological and microevolutionary processes. Mol Ecol.

[32] Gower CM, Gabrielli AF, Sacko M, Dembele R, Golan R, Emery AM (2011). Population genetics of *Schistosoma haematobium*: development of novel microsatellite markers and their application to schistosomiasis control in Mali. Parasitology.

[33] Aemero M, Boissier J, Climent D, Moné H, Mouahid G (2015). Genetic diversity, multiplicity of infection and population structure of *Schistosoma mansoni* isolates from human hosts in Ethiopia. BMC Genet.

[34] Liang YS, Coles GC, Doenhoff MJ, Southgate VR (2001). *In vitro* responses of praziquantel-resistant and -susceptible *Schistosoma mansoni* to praziquantel. Int J Parasitol.

[35] William S, Sabra A, Ramzy F, Mousa M, Demerdash Z, Bennett JL (2001). Stability and reproductive fitness of *Schistosoma mansoni* isolates with decreased sensitivity to praziquantel. Int J Parasitol.

