# Supplementary figures and images for: Phenotypic and genotypic monitoring of Schistosoma mansoni in Tanzanian schoolchildren five years into a preventative chemotherapy national control programme
Source: Parasit Vectors. 2017 Dec 2;10:593. doi: 10.1186/s13071-017-2533-6 (PMC5712074; doi:10.1186/s13071-017-2533-6)

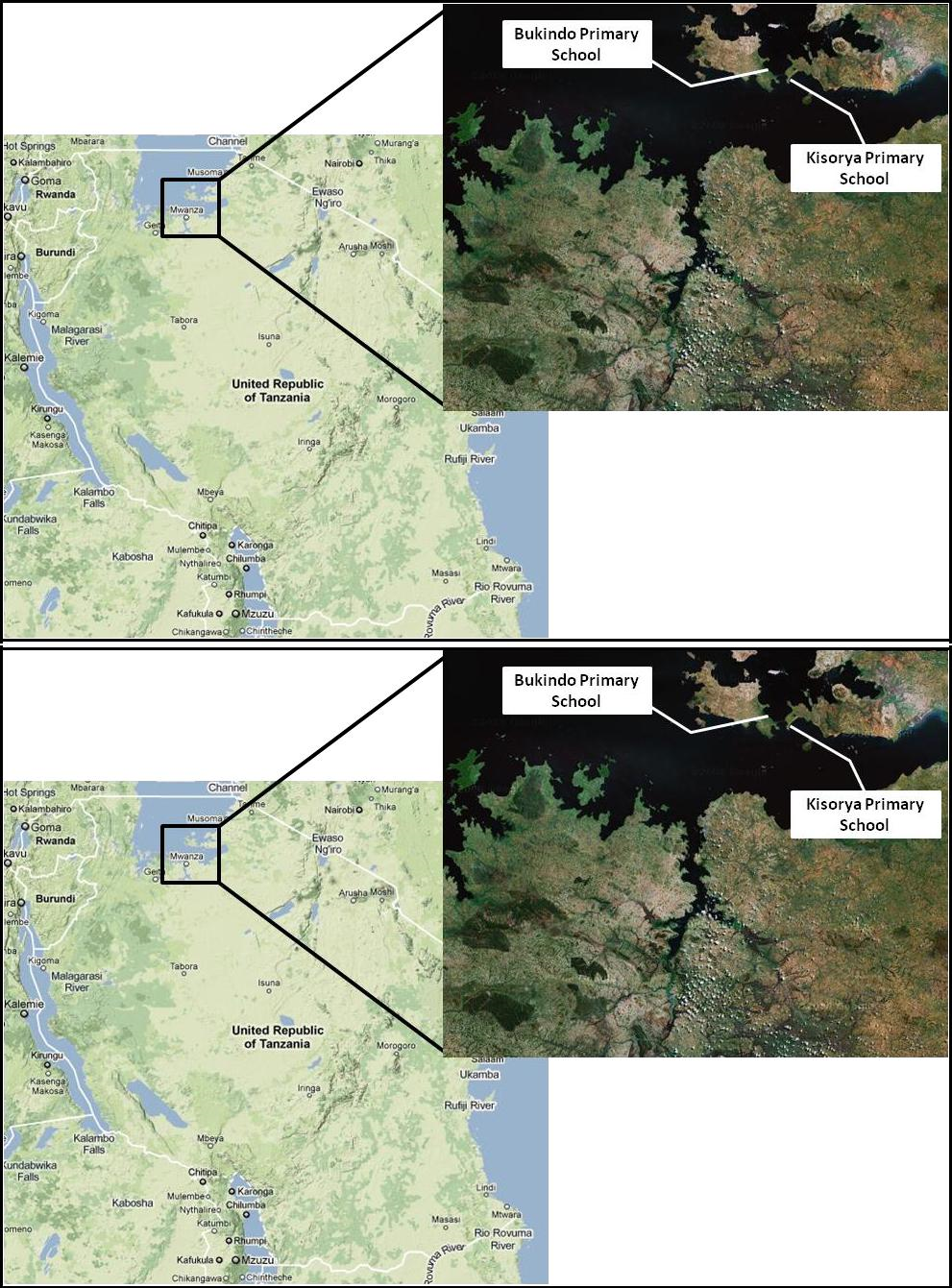

Supplement: Additional file 1: Figure S1. — Location of study sites in Tanzania. In order to collect S. mansoni miracidia, 7–11 year old children from two schools of Tanzania’s highly endemic Lake Victoria region were sampled. Bukindo Primary School is situated on Ukerewe Island inside Lake Victoria (Ukerewe District, Mwanza Region). The second school studied was Kisorya Primary School (Bunda District, Mara Region) which is located on the mainland. Both schools are in close proximity to the lakeshore. (TIFF 1924 kb) [file 13071_2017_2533_MOESM1_ESM.tif]
